# 

**Published:** 1870-02

**Authors:** 


### CONTENTS.

ORIGINAL COMMUNICATIONS.

Os-Artificial Vindicated .......... 51
Chemical Essays .......... 55
Miscellanies .......... 59
A Visit .......... 65

PROCEEDINGS OF SOCIETIES.

Fourth Annual Meeting of the Ohio State Dental Society .......... 70
Effects of Copper on the System .......... 92

SELECTIONS.

Aluminum—Its Mode of Working, and Alloys .......... 93
The New Metals .......... 96
The Spectroscope .......... 98
A Forger of Diplomas Caught .......... 99
Green Line on the Gum from Copper Poisoning .......... 100
The Unwearied Action of the Heart .......... 101
Agents for the Removal of Stains .......... 101
Popular Ignorance of Medicine .......... 102
Female Medical Education .......... 102
Paste .......... 102
Sleep as a Poison .......... 103
Conservative Surgery .......... 105
A Course of Practical Chemistry .......... 106

EDITORIAL.

Legal .......... 107

